# A RAC/CDC-42–Independent GIT/PIX/PAK Signaling Pathway Mediates Cell Migration in *C. elegans*

**DOI:** 10.1371/journal.pgen.1000269

**Published:** 2008-11-21

**Authors:** Mark Lucanic, Hwai-Jong Cheng

**Affiliations:** 1 Center for Neuroscience, University of California Davis, Davis, California, United States of America; 2 Cell and Developmental Biology Graduate Group, University of California Davis, Davis, California, United States of America; 3 Department of Neurobiology, Physiology and Behavior, College of Biological Sciences, University of California Davis, Davis, California, United States of America; 4 Department of Pathology and Laboratory Medicine, School of Medicine, University of California Davis, Davis, California, United States of America; Stanford University Medical Center, United States of America

## Abstract

P21 activated kinase (PAK), PAK interacting exchange factor (PIX), and G protein coupled receptor kinase interactor (GIT) compose a highly conserved signaling module controlling cell migrations, immune system signaling, and the formation of the mammalian nervous system. Traditionally, this signaling module is thought to facilitate the function of RAC and CDC-42 GTPases by allowing for the recruitment of a GTPase effector (PAK), a GTPase activator (PIX), and a scaffolding protein (GIT) as a regulated signaling unit to specific subcellular locations. Instead, we report here that this signaling module functions independently of RAC/CDC-42 GTPases in vivo to control the cell shape and migration of the distal tip cells (DTCs) during morphogenesis of the *Caenorhabditis elegans* gonad. In addition, this RAC/CDC-42–independent PAK pathway functions in parallel to a classical GTPase/PAK pathway to control the guidance aspect of DTC migration. Among the *C. elegans* PAKs, only PAK-1 functions in the GIT/PIX/PAK pathway independently of RAC/CDC42 GTPases, while both PAK-1 and MAX-2 are redundantly utilized in the GTPase/PAK pathway. Both RAC/CDC42–dependent and –independent PAK pathways function with the integrin receptors, suggesting that signaling through integrins can control the morphology, movement, and guidance of DTC through discrete pathways. Collectively, our results define a new signaling capacity for the GIT/PIX/PAK module that is likely to be conserved in vertebrates and demonstrate that PAK family members, which are redundantly utilized as GTPase effectors, can act non-redundantly in pathways independent of these GTPases.

## Author Summary

Cell migration is essential for the development and maintenance of metazoan tissue. A migrating cell must navigate through complex environments and properly interpret the signals present in its path. This cellular movement is accomplished through transduction of the signals into directed reorganization of the cellular structure. Among the most important molecules that orchestrate signals from the exterior of the cells into cellular movement are the small GTPases, which function in intracellular signal transduction cascades. We have studied the interactions between GTPases, their effectors, and the environmental signals during cellular migrations in *C. elegans*. We have found that while some GTPases do control the guidance of these migrating cells, a certain highly conserved complex of proteins thought to be involved in mediating GTPase signaling during cellular migrations in fact functions independently of these GTPases to specifically control the structure and movement of the migrating cells. These results have revealed an unexpected role of a well-known and highly conserved signaling complex, which is particularly important since members of this complex are associated with human mental retardation. Our results may imply that the disease phenotype is likely more complex than previously thought and may in fact occur from disruption of this novel pathway.

## Introduction

The GIT/PIX/PAK signaling pathway is a highly conserved signaling module which controls cytoskeletal dynamics across metazoans. The functions of this signaling complex are diverse. In humans it controls the migrations of fibroblasts through modulation of adhesion complexes, and participates in T cell receptor signaling in the immune system. The GIT/PIX/PAK complex has also been shown to regulate neuronal plasticity and development in the nervous system [1]–[3]. The importance of this protein complex is further highlighted by the observation that in humans a loss of either PAK3 or αPIX leads to impaired function of the nervous system from nonsyndromic mental retardation [4],[5]. To further understand how this complex functions in a well-defined in vivo system, we have isolated the *C. elegans* orthologs of the GIT/PIX/PAK complex and studied their roles in the migrations of the gonad distal tip cells (DTCs).

PAKs are downstream effectors of RAC and CDC-42 GTPases [6]. RAC and CDC-42 are RAS superfamily GTPases of the RHO subtype and are known to control cytoskeletal dynamics through their function as molecular switches [7]. In the canonical GTPase/PAK pathway, an activated RAC or CDC-42 GTPase binds to PAK and stimulates the activation of PAK's kinase activity. Despite the importance of the canonical GTPase/PAK pathways it has become increasingly clear that PAKs can also function in non-canonical pathways independent of GTPases [8]. While studies in vertebrates have indicated the likely existence of GTPase-independent PAK activation pathways the mechanistic details, biological relevance and prevalence of these pathways remain poorly understood.

GIT and PIX have been shown to regulate cellular processes through PAKs in diverse model systems [2],[3],[9]. It is generally thought that GIT/PIX/PAK pathways utilize GTPases, as PIX contains a clear GEF (guanine exchange factor) domain for RAC and CDC-42 GTPases and all of these proteins control the same cellular processes. Recently two reports have indicated a possible GTPase-independent GIT/PIX/PAK signaling pathway is likely to exist. These studies found in vitro that PAK can be activated by PIX and GIT in the absence of a GTPase-PAK interaction. In the first of these studies it was shown that a guanine exchange factor (GEF) deficient PIX can activate PAK, while the second study demonstrated that the ARF GAP (ADP-ribosylation factor GTPase activating) domain of GIT can activate PAK [10],[11]. These two studies suggested that the GIT/PIX/PAK complex can function independent of GTPases but the possible in vivo function of this pathway remains unclear.

We find that in *C. elegans* the PAKs, RACs, CDC-42, GIT and PIX are all involved in gonad morphogenesis. During gonad development the DTC functions as a leader cell to direct its elongation [12]–[14]. The movement of the DTC is controlled by guidance molecules [12],[15],[16], as well as other factors that are associated with the formation and regulation of the extracellular matrix (ECM) [17]–[22]. However, little is known about the signaling pathways that transduce these environmental cues into directed cell movements. Here we define two distinct signaling pathways that control the guidance of the DTCs during gonad morphogenesis. One is a typical GTPase/PAK pathway that utilizes either PAK redundantly while the other is a GIT/PIX/PAK pathway that also controls the shape and migration of the DTCs. Remarkably we find that the highly conserved GIT/PIX/PAK complex is specific for one of the PAKs and functions in a novel RAC/CDC-42 independent manner during these processes.

## Results

### Two *C. elegans* PAKs, PAK-1 and MAX-2, Are Partially Redundant for Gonad Morphogenesis

**Figure 1:**
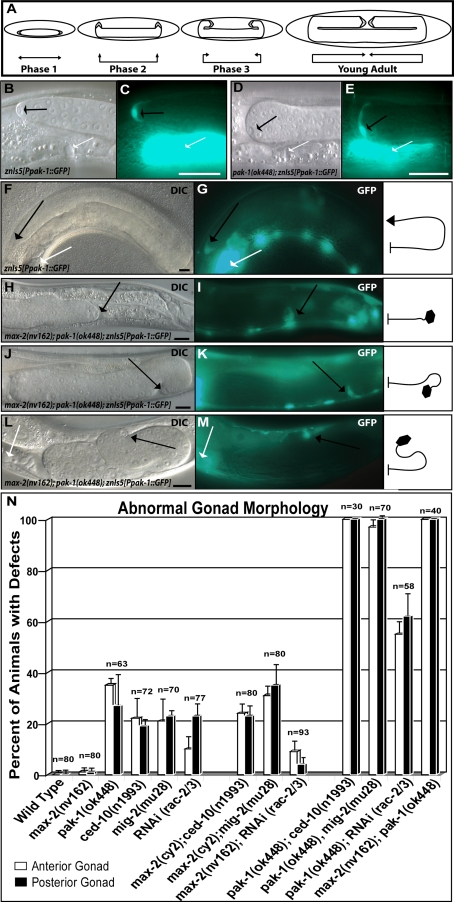
RAC-dependent and RAC-independent PAK pathways control gonad morphogenesis. (A) A diagram of gonad elongation highlighting the three phases of DTC migration. (B–C) High magnification image of a wild-type distal tip cell from in the DTC reporter background (*znIs5[Ppak-1::GFP]*) showing the tapering morphology of a wild type DTC. (D–E) High magnification image of a *pak-1* mutant showing the bloated DTC. (F–M) The posterior gonad arms of wild-type and *max-2;pak-1* double mutants in a DTC reporter background (*znIs5[Ppak-1::GFP]*). DIC images (F,H,J,L) and fluorescence images (G,I,K,M) show the morphology of the gonad and the DTC, which are summarized as a schematic diagram in the right box. In wild-type animals (F–G), the posterior gonad is long and U shaped, and tapers sharply at the DTC (triangle in the diagram). *max-2;pak-1* mutants show defects in all phases of DTC guidance. Shown here are three examples: the DTC fails to make the dorsal turn and has stalled after the first phase of migration (H–I); the DTC has made an incorrect turn at the phase 2/3 transition resulting in a question mark shaped gonad (J–K); the DTC makes abbreviated migrations during the first two phases of migration and made an extra turn prior to finishing its migration (L–M). In all cases, the DTCs have an abnormal morphology (hexagon in the diagram). White arrow, vulva; black arrow, DTC. Scale bar: 10 micrometers except in B and E where the scale bar is 25 micrometers. (N) A graphical representation of the defects in DTC morphology, migration or guidance (collectively referred to as gonad morphology) of *pak* and *rac* mutants. The bars represent the standard error of the mean and n = the number of both anterior and posterior gonads scored.

While investigating the roles of the *C. elegans* PAKs we found that the two PAKs, *pak-1* and *max-2* are redundantly required for proper formation of the gonad. In wild type animals the two DTCs function as leader cells to guide the elongating gonads, which eventually form two bilaterally symmetric U shaped gonad arms (Figure 1A). The elongation of gonad during morphogenesis occurs in three phases (Figures 1A–C). In the first phase, the DTC leads the developing gonad away from a mid-body position along the ventral side of the animal. In the second phase the DTCs turn orthogonally and migrate towards the dorsal side of the animal. In the third and final phase, the two DTCs turn back and then migrate towards each other, reaching the vulva by the young adult stage. Throughout gonad elongation the DTCs exhibit a sharp tapering morphology such that they have a cone-like shape when viewed from the side (Figures 1B–C). In order to understand the role of PAK signaling pathways in gonad morphogenesis, we first examined individual *pak* mutants.

**Figure 2:**
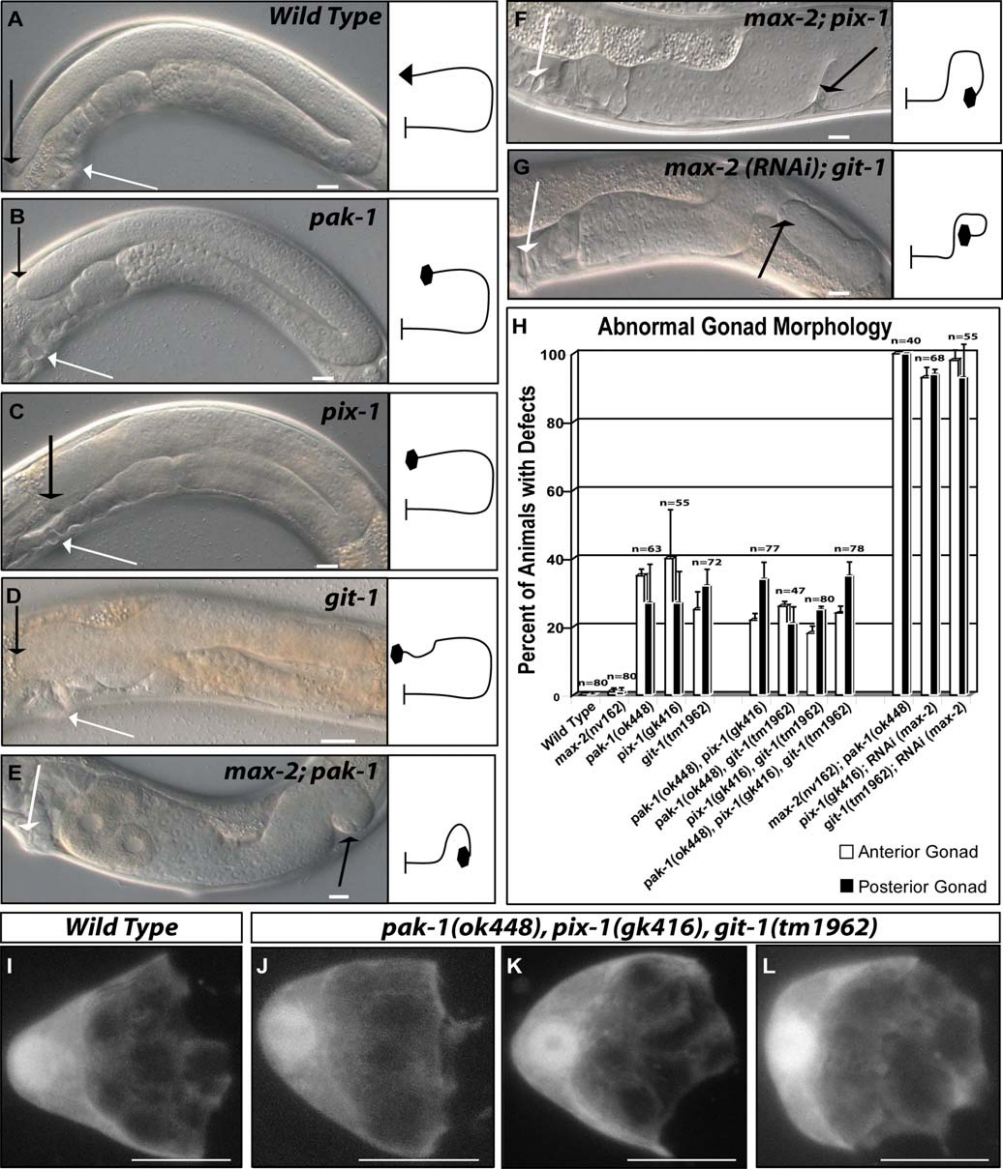
*pak-1*, *pix-1* and *git-1* act together to control the migration and morphology of DTCs and in parallel to *max-2* to mediate DTC guidance. (A–E) DIC images of the posterior gonads of young adult animals. Wild-type animals (A) have a U shaped half gonad that extends proximally from the vulva (white arrow) and distally to the DTC (black arrow). Wild-type distal gonads generally extend slightly past the vulva and end in a point that tapers into the DTC. In *pak-1* (B), *pix-1* (C) and *git-1* (D) mutants the DTCs often fail to extend all the way to the vulva (B,C) and have bloated distal gonads (B,C,D). In *pak-1*; *max-2* (E), *pix-1*; *max-2* (F) and *git-1*; RNAi *(max-2)* (G) double mutants major DTC guidance and elongation defects are observed in addition to the bloated distal gonad defect. The distal tip cells of these double mutants make a variety of improper turns including ventral turns (E,F), ectopic dorsal turns (F,G) and improper turns away from the midbody (E,F,G). Double mutants of *pix-1(gk416)* or *git-1(tm1962)* with *max-2* generally rupture at the vulva in the adult stage. Escapers of the rupture phenotype fail to yield offspring. Scale bar: 10 micrometers. (H) Graphical representation of the percent of animals of a given genotype that were found to have defects in DTC morphology, migration or guidance (collectively referred to as gonad morphology). The bars represent the standard error of the mean and n = the number of both anterior and posterior gonads scored. (I–L) Projections through a Z-stack taken with confocal microscopy of wild type (I) or mutant (J–L) animals expressing mRFP (from a *lag-2* promoter) shows the respective morphology of their migrating DTCs.

*pak-1* mutants were found to display mild defects in DTC morphology and migration (Figures 1D–E and 2B). In *pak*-1 mutants the DTCs generally lacked the sharp tapering morphology of wild type DTCs and instead had a bloated or distended structure (morphology defect) (Figures 1D–E). The *pak-1* mutant DTCs also often failed to migrate all the way to the vulva (migration defect) (Figure 2B). *max-2* mutants did not exhibit any of these defects. To reveal redundancy between these genes we examined PAK double mutants for gonad defects. The *pak-1* and *max-2* mutants used are putative null alleles [23]. The *pak-1(ok448)* allele has a deletion that removes most of the kinase domain, results in a frame shift and introduces an early stop codon. The *max-2* allele *nv162* has a deletion that removes the start codon, the first 4 exons and does not contain another in frame start codon until midway through the kinase coding sequence. The *max-2(cy2)* allele contains a missense mutation resulting in a glycine to glutamate substitution at a conserved residue in the kinase domain. The *pak-1;max-2* double mutants exhibit even more severe defects than *pak-1* single mutants. In addition to morphology defects, the DTCs in *pak-1;max-2* double mutants wandered during their migrations (guidance defect), failed to execute at least one of the turns and did not migrate completely to the vulva (Figures 1H–M). These results demonstrate a role for the PAKs in regulating DTC morphology, migration and guidance during gonad morphogenesis, and suggest that the two PAKs are only partially redundant, such that there is a role for PAK-1 in regulating DTC morphology and migration that MAX-2 does not fulfill.

### MAX-2 Works with the RAC GTPases, While PAK-1 Functions at Least Partly in Parallel

PAKs are the best known RAC GTPase effectors. There are three *rac* genes in *C. elegans*: *ced-10*, *mig-2* and *rac-2/3* [24]. The RACs themselves are required for DTC guidance, and they are partially redundant with each other for gonad development [24],[25]. We therefore investigated whether the PAKs act with the RACs in DTC guidance. We made use of the following *rac* mutants: for *mig-2* we utilized the putative null allele *mig-2(mu28)*. As CED-10 is required for embryogenesis, we utilized the *ced-10(n1993)* allele which is expected to be a strong loss of function. Because of the presence of the gene duplication in *rac-2/3* we utilized RNAi for the *rac-2/3* loss of function analysis. As previously reported we observed characteristic extra turns during the last phase of the DTC migrations resulting from a loss of function in any of the *racs* (Figure 1N). We then examined double mutants of the two *paks (pak-1* and *max-2)* with the *racs*. Mutations in *max-2* did not enhance the DTC guidance defects of any of the *racs* (Figure 1N), indicating that MAX-2 works with the RACs in DTC guidance. In contrast, *pak-1* mutants severely enhanced the DTC guidance defects of any of the *rac* mutants (Figure 1N), indicating that PAK-1 acts at least partly in parallel to the RAC GTPases.

### GIT-1, PIX-1, and PAK-1 Work Together to Control DTC Migration and Morphology

To identify factors that may function with PAK-1 in the RAC-independent pathway, we examined genes that are known to interact with PAKs in other species. In this manner we identified orthologs of vertebrate PIX and GIT genes, which are referred to as *pix-1* and *git-1* respectively. PIX and GIT proteins are highly conserved among worms, flies, mice and humans (Figure S1). Utilizing putative promoter regions from the two genes to drive GFP expression in *C. elegans* we studied their expression patterns and found that both genes were expressed in the DTCs throughout the DTC migrations. (Figure S1). To begin to address the functions of *pix-1* and *git-1* in the DTCs we examined deletion mutants for gonad defects. The allele *pix-1(gk416)* has a deletion beginning 4 codons after the translational start, which removes the entire SH3 domain and is expected to result in a very early stop codon due to a frame shift. The nature of this deletion indicates that *gk416* is a null allele. For *git-1* we utilized *git-1(tm1962)* which contains a 484 bp genomic deletion (in frame) resulting in 133 amino acids of the protein being deleted including the second GIT domain. As this domain is required to bind PIX in fibroblasts [9], any functional protein generated in the mutant is expected to be unable to bind PIX-1. The *tm1962* deletion likely results in a strong loss of function.

Similar to *pak-1* mutants, the *pix-1* and *git-1* mutants exhibited the characteristic defects in DTC migration and DTC morphology (Figures 2A–D), but not the DTC guidance defects seen in the *rac* single mutants or in the *pak-1;max-2* double mutants. We then tested whether *pak-1*, *pix-1* and *git-1* function together in a pathway by examining all possible double mutant combinations. Double mutant combinations of *pak-1*, *pix-1* and *git-1* did not enhance the DTC defects relative to the strongest single mutant (Figure 2H). Nor was there any statistical difference in DTC defects between the triple mutant and any of the single mutants (Figure 2H). Interestingly, the characteristic morphology defects found in the single, double or triple mutants of *pak-1*, *pix-1* or *git-1* could be observed in actively migrating DTCs (compare Figure 2I with 2J–L). Collectively these data indicate that GIT-1/PIX-1/PAK-1 signaling complex is required for the proper migration of the DTCs and the regulation of their cellular morphology and this pathway is not redundant with the classical RAC/PAK pathway.

**Figure 3:**
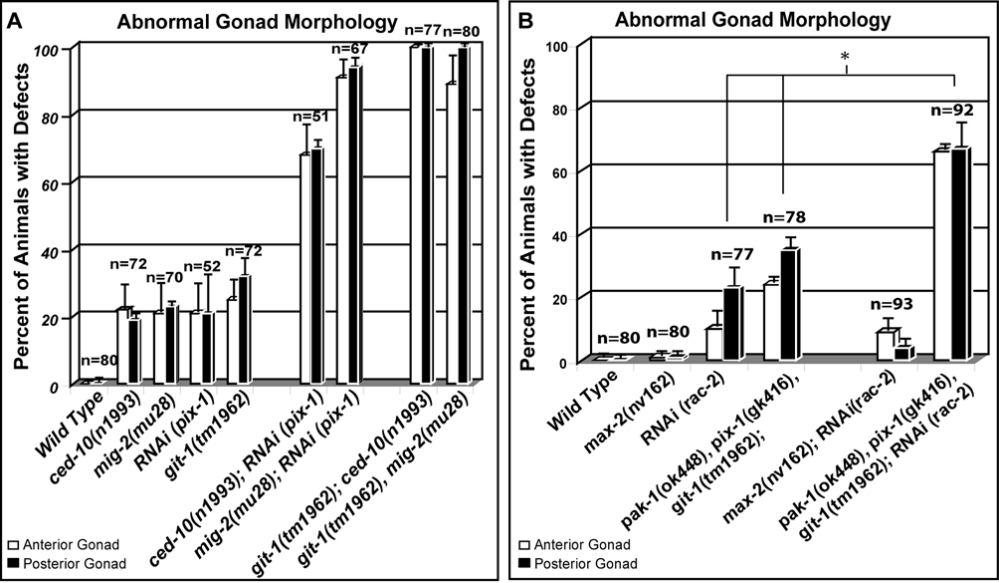
*pak-1*, *pix-1* and *git-1* function in a pathway and act in parallel to the *racs* to control gonad morphogenesis. Double mutants of *pak-1*, *pix-1* or *git-1* with any of the *racs* cause major defects in the guidance, migration and morphology of the DTCs. (A) Graphical representation of the percent of animals of a given genotype that were found to have defects in DTC morphology, migration or guidance. As we were unable to isolate progeny of *pix-1;rac* homozygotes we utilized RNAi to assay enhancement of DTC migration defects in (B). (B) Graphical representation of the results from RNAi of the *rac-2/3* gene, demonstrating that a loss of *rac-2/3* results in a significant enhancement of the *pak-1*, *pix-1*, *git-1* triple mutant. However, as with the other *racs*, *max-2* null mutants do not significantly enhance the guidance defects resulting from RNAi of *rac-2/3*. Graphs represent combined defects from scoring DTC morphology, migration or guidance (collectively referred to as gonad morphology). The bars represent the standard error of the mean and n = the number of both anterior and posterior gonads scored. The asterisks represent significant differences P<0.001.

In contrast, MAX-2 functions in parallel to PIX-1 and GIT-1 to mediate DTC guidance. In addition to the morphology and migration defects of the GIT/PIX/PAK pathway, double mutants of *max-2* with any of the genes from this pathway (*pak-1*, *pix-1* or *git-1*) also showed major guidance defects in all stages of gonad elongation (Figures 2E–H). These results indicate that PAK-1, PIX-1 and GIT-1 function in a redundant DTC guidance pathway in parallel to MAX-2. As MAX-2 works with the RAC GTPases this suggests that GIT-1 and PIX-1 may also function independent of the RACs. In support of this, *pix-1* and *git-1* mutants also profoundly enhance the guidance defects resulting from a loss of function in the *rac* genes (Figure 3). The gonadal defects seen in *pix-1;rac* and *git-1;rac* double mutants were similar to the *pak-1;rac* double mutants, which in turn were similar to the double mutants of the *git-1/pix-1/pak-1* pathway with *max-2*.

In summary, our mutant analysis showed that any mutant in the GIT-1/PIX-1/PAK-1 pathway led to migration and morphology defects of the DTCs, while a loss of any of the *racs* (which work in a pathway with *max-2*) led to guidance defects of the DTCs. Double mutants between these pathways led to severe defects in DTC guidance. Taken together, these results indicate that there are at least two distinct PAK pathways controlling DTC guidance during gonad morphogenesis: one is a classical RAC/PAK pathway, in which both MAX-2 and PAK-1 are utilized. The other is RAC-independent PAK pathway, in which PAK-1 (but not MAX-2), PIX-1 and GIT-1 are utilized and this latter pathway is used non-redundantly to regulate DTC migration and morphology.

### The GIT-1/PIX-1/PAK-1 Pathway Functions Independent of RAC and CDC-42 GTPases

**Figure 4:**
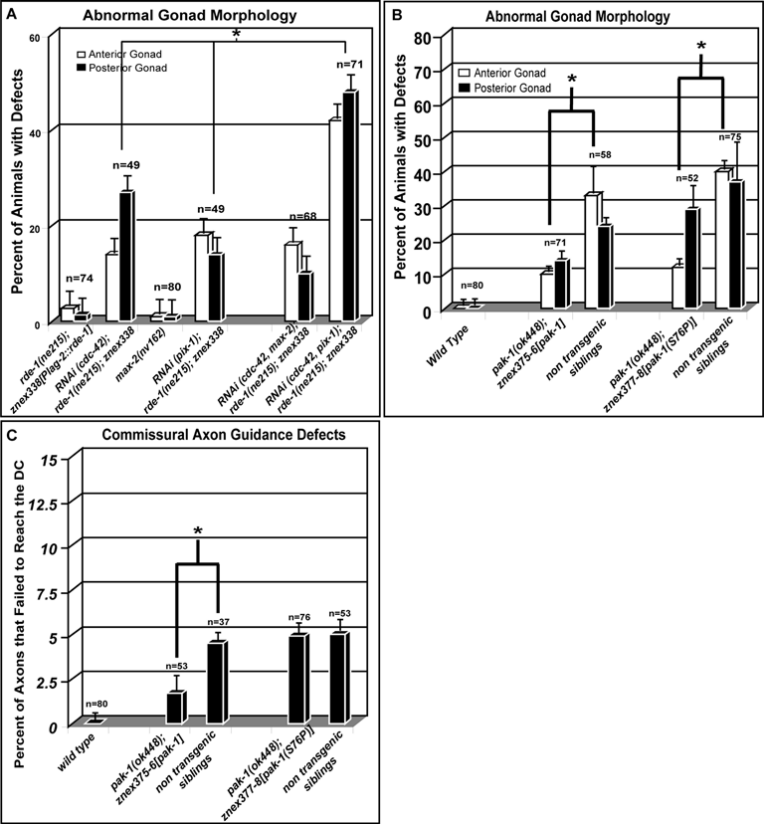
The GIT/PIX/PAK pathway mediates DTC migration and morphology independent of GTPases. (A) A graphical representation of defects in DTC morphology, migration or guidance (collectively referred to as gonad morphology) in the HJ229 strain by itself, and after injection of dsRNA from candidate genes. The genotype of HJ229 is *rde-1(ne215)*; *znex338[Plag-2::rde-1*, *Plag-2::mRFP]*. RNAi of *cdc-42* in the HJ229 background results in strong defects. These defects are significantly enhanced by addition of *pix-1* dsRNA but not by addition of *max-2* dsRNA. (B–C) The GTPase binding domain of PAK-1 is dispensable for gonad morphology but not for commissural motor axon guidance. Transgenics expressing wild type, *pak-1* or *pak-1(S68P)* (a *pak-1* gene with a point mutation in a conserved residue required for binding GTPases) are all significantly rescued for the anterior gonad morphology, while there is a non statistically significant trend towards rescuing the posterior defect (B). Wild-type PAK-1 rescues the GTPase axon guidance activity while the GTPase binding mutant does not (C). The experiment was performed in the *max-2* RNAi background to enhance the *pak-1* defect. The error bars represent the standard error of the mean and n = the number of animals scored. The asterisks represent significant differences based on the students test (P<0.05).

Since the GIT/PIX/PAK pathway functions independent of RAC GTPases, we next sought to explore whether the pathway functions independent of other GTPases. CDC-42 is also a RHO subfamily GTPase that has been shown to activate PAKs, and PIX is predicted to also be a GEF (Guanine Exchange Factor) for CDC-42. If the GIT/PIX/PAK pathway does function independent of CDC-42, knocking down CDC-42 would enhance the defects resulting from a loss of the GIT/PIX/PAK pathway. As CDC-42 is required for viability, we utilized tissue specific RNAi [26] in the post-embryonically born DTCs, to bypass the embryonic requirement for CDC-42. Double RNAi of *cdc-42* and *pix-1* caused much more profound defects than RNAi of either of them alone. However, RNAi of *max-2* did not enhance the defects caused by RNAi of *cdc-42* (Figure 4A). This data indicates that the GIT/PIX/PAK pathway may function independently of CDC-42. Interestingly the lack of enhancement with the *cdc-42*; *max-2* double RNAi may suggest that MAX-2 works with CDC-42 during gonad elongation. However these negative results are less than definitive as RNAi causes a partial loss of function and simply may not cause enough of a knock down to generate any possible enhancement of the *cdc-42* phenotype.

If PAK-1 functions independently of CDC-42 during DTC migrations, PAK-1 may not require conserved amino acids that allow it to bind GTPases. To test this we selectively altered PAK-1 at an amino acid in the GTPase binding domain *(pak-1(S76P))* that in other systems has been shown to be required for binding to CDC-42 and that is likely to disrupt binding to all GTPases [11]. We found that both wild type and the mutant PAK-1 partially rescued the *pak-1* gonad morphology defects (Figure 4B). This suggests that activation by CDC-42 is not necessary for the non redundant PAK-1 function in DTC morphology and migration. As an important control, we tested *pak-1(S76P)* in the guidance of motor axons, where we showed previously that PAK-1's function is RAC dependent [23]. As expected, injecting *pak-1(S76P)* failed to rescue the axon guidance defect in *pak-1* mutant, while injecting wild-type *pak-1* gene did (Figure 4C). This latter result also indicates that the mutated PAK-1 loses its ability to interact with RAC GTPases. Collectively our results demonstrate that the GIT-1/PIX-1/PAK-1 pathway functions at least partly independent of RAC and CDC-42 GTPases.

### GIT-1 and PIX-1 Function Cell Autonomously and Co-Localize in Migrating DTCs

**Figure 5:**
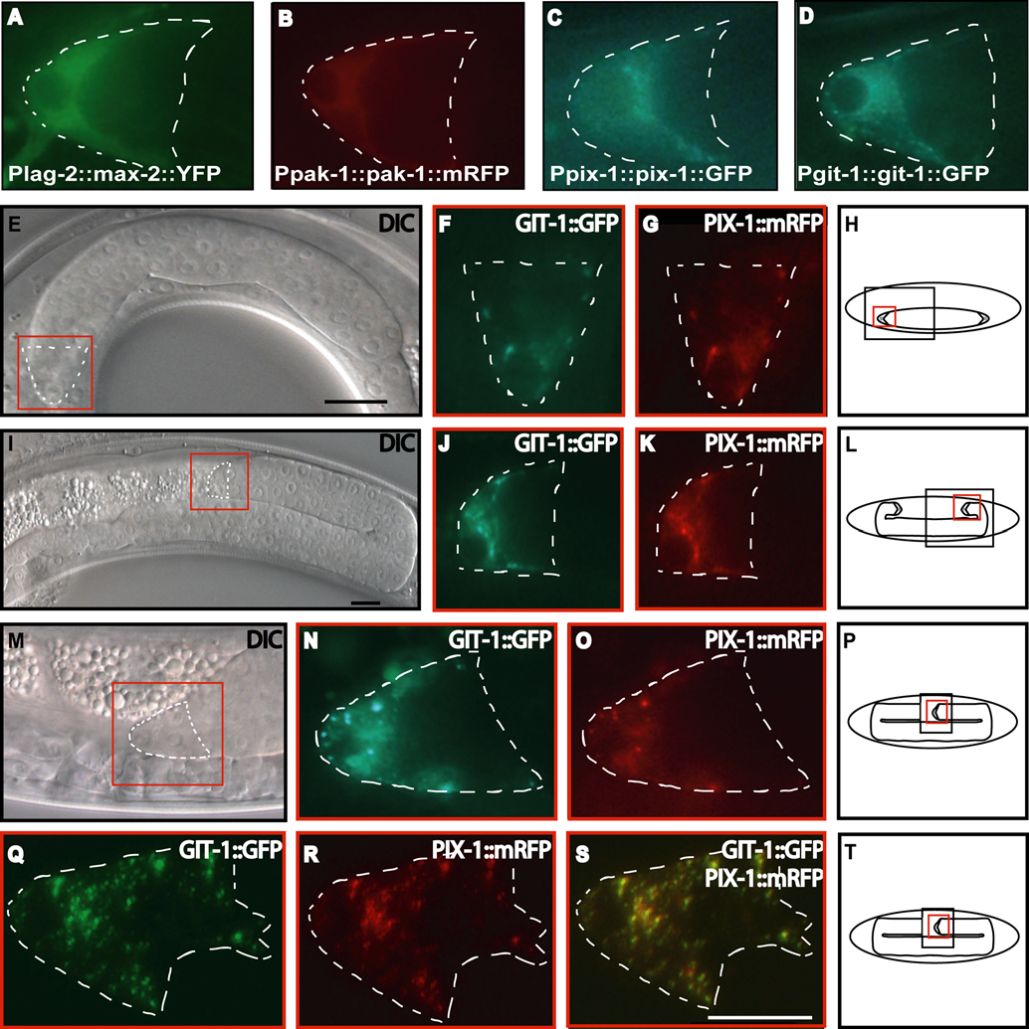
PIX-1 and GIT-1 co-localize in migrating distal tip cells. (A–D) Fluorescence images of tagged proteins in late L4 distal tip cells. The dotted lines define the border of the DTC. The subtext in the images describes the transgene. MAX-2::YFP (A) and PAK-1::mRFP (B) were always found to be diffusely localized throughout the cytoplasm, while both PIX-1::GFP (C) and GIT-1::GFP (D) localized in a punctate pattern. (E–T) DIC (E,I,M) and fluorescence images (F,G,J,K,N,O,Q,R,S) and correlating diagrams (H,L,P,T) of gonad arms and distal tip cells demonstrating the phase of migration and localization of tagged proteins. The transgenics are co-expressing GIT-1::GFP and PIX-1::mRFP each under it own promoters. (E–H) show phase 1 of migration, (I–L) show early phase 3 and (M–T) show a later point in phase 3. (Q–S) is a collapsed projection through a Z-stack at the end of phase 3. (S) shows a merged image of the green and red channels demonstrating that the signals have nearly complete overlap. In all images anterior is to the left and the scale bar is 10 micrometers.

To gain insight into these distinct pathways controlling gonad morphogenesis, we used fluorophore tagged proteins to examine the subcellular localizations of the PAKs, PIX and GIT in migrating DTCs in vivo. These tagged proteins rescued the DTC defects when expressed in the DTCs of the respective mutants (Figure S2). We found that the tagged PAKs were diffusely present throughout the cytoplasm of the DTC during all stages of its migration (Figure 5A–B) suggesting that the PAKs function through a transient local activation mechanism. In contrast, both GIT-1::GFP and PIX-1::GFP localized to punctate structures in the DTC during its migrations (Figure 5C–D). These puncta were observed throughout the cytoplasm of the migrating DTC. In addition, we found that GIT-1::GFP and PIX-1::mRFP co-localize throughout all of the phases of the migrating DTC (Figure 5E–T). The extent of co-localization is nearly complete as there were few, if any, sites in the DTC where the RFP and GFP signals did not overlap (Figure 5Q–T). These results indicate that *C. elegans* GIT-1 and PIX-1 are likely to interact directly, as has been repeatedly observed for their orthologs in a variety of different systems [1],[2],[27]. The punctate pattern is highly reminiscent of the localization of GIT/PIX in other systems where they have been characterized as forming large multimeric complexes that are thought to be scaffolds for intracellular signaling [28]. Collectively our results suggest that the GIT/PIX complex locally activates PAK-1 from a reservoir of cytoplasmically localized inactive PAK-1.

### Both the RAC/CDC-42 GTPase–Dependent and –Independent PAK Pathways Likely Function to Mediate Integrin Signaling

**Figure 6:**
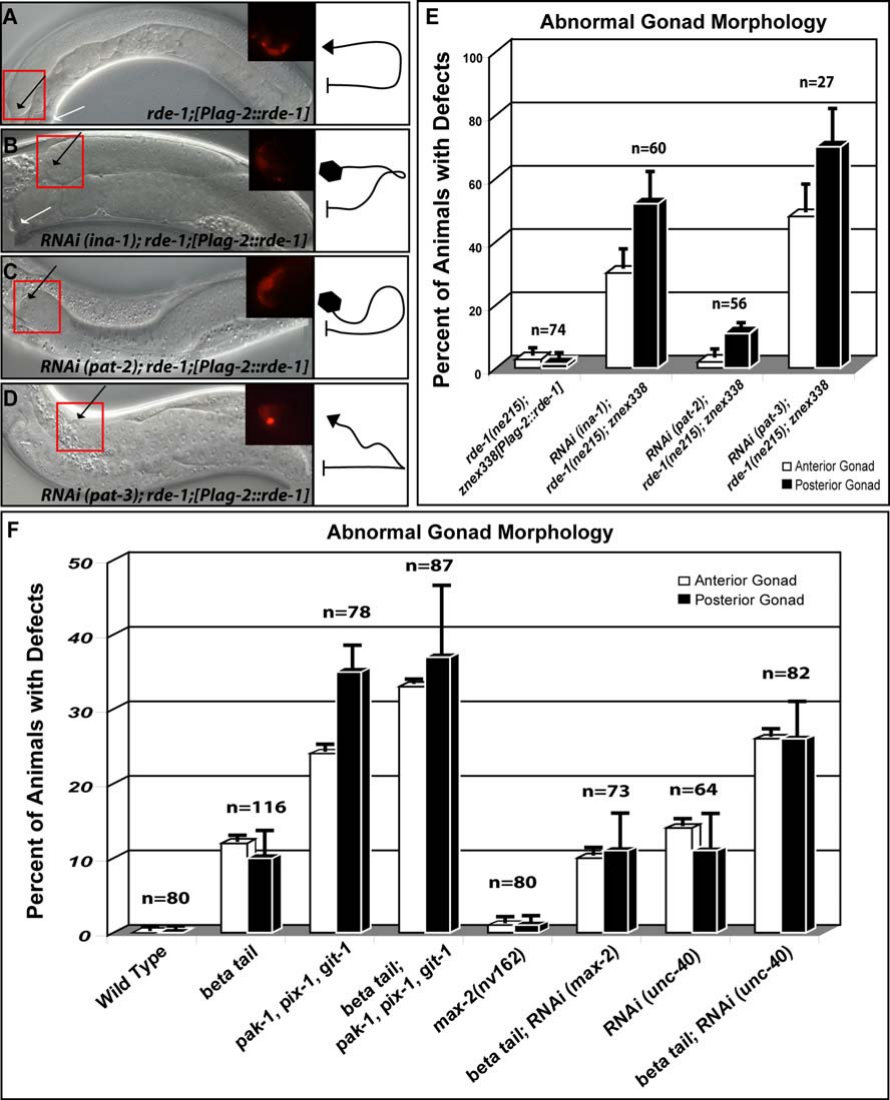
Integrins likely function with the GIT/PIX/PAK and the RAC/PAK signaling pathways to control gonad morphogenesis. (A–D) Representatives images of the posterior gonad in of the transgenic strain HJ229 which contains *rde-1(ne215)*; *znex338[Plag-2::rde-1*, *Plag-2::mRFP]* either wild type (A) or with tissue specific RNAi for *ina-1* (B), *pat-2* (C), or *pat-3* (D). All images are from a similar phase of DTC migration. The inset image, taken from the boxed region in DIC, shows the mRFP signal which diffusely labels the cytoplasm of the DTC but often forms aggregates after migration. The morphology of the gonad and the DTC is summarized as a schematic diagram in the right box. In HJ229 the gonad is U shaped and tapers at the DTC (A). Representative image from RNAi of *ina-1*, resulting in DTC guidance and morphological defects such as precocious dorsal turns, a failure to extend all the way to the vulva and a bloated distal gonad (B). Representative image from RNAi of *pat-2*, resulting in guidance defect of ventral turn during phase 3 as well as bloated distal gonad and distended DTC (C). Representative image from RNAi of *pat-3* showing DTC guidance and migration defect in phase 2 resulting in a triangular shaped gonad (D). (E) Graphical representation of the defects in DTC morphology, migration or guidance (collectively referred to as gonad morphology) resulting from the tissue specific RNAi of the integrin subunits. (F) Graphical representation of the defects in DTC morphology, migration or guidance (collectively referred to as gonad morphology) resulting from expressing an interfering *pat-3* beta integrin construct (beta tail). The experiments were performed such that the *beta tail* and the *beta tail*; *pak,pix,git* mutant were scored as first cousins. For the *unc-40* experiments the RNAi (*unc-40*) and the *beta tail*; RNAi (*unc-40*) animals were non-transgenic and transgenic siblings respectively. The same two independently generated transgenic lines were used for both experiments. The bars represent the standard error of the mean and n = the number of both anterior and posterior gonads scored.

PAKs, PIX, GIT and RACs have all been implicated in integrin-regulated processes in other model systems [9],[29]. To explore whether integrin signaling in the DTC is mediated by PAK signaling pathways, we first examined the phenotypes of integrin mutants by RNAi. Integrins function as heterodimers that consist of alpha and beta subunits. *C. elegans* genome contains two alpha (*ina-1* and *pat-2*) and a single beta (*pat-3*) subunits. The integrins have previously been implicated in controlling DTC migration [15],[30],[31]. As all of the integrin genes are required for embryogenesis, we examined their function in DTCs with tissue specific RNAi. We found that a loss of function in any of the integrin genes led to similar defects as those we observed in the double PAK pathway mutants. The integrin mutants have both the severe migration and guidance defects of *(rac/max-2);(pak-1/pix-1/git-1)* double mutants as well as the bloated DTC morphology phenotype observed in mutants of the GIT-1/PIX-1/PAK-1 pathway (Figures 6A–E). This was also observed in the two available *ina-1* hypomorphs *gm39* and *gm144* (data not shown). Unfortunately we were unable to generate double mutants of these hypomorphs with either of the *paks*, leading us to conclude that these double mutants may be unviable. Nevertheless, these data suggest that the integrins may function with both the GIT/PIX/PAK and the RAC/PAK signaling pathways.

To further determine whether the PAK signaling pathways function with the integrins, we made use of a PAT-3 beta-integrin interfering construct (beta tail) previously reported to disrupt integrin signaling in the DTCs [30]. If the PAK pathways function with the integrins, a loss of either PAK pathway may not lead to an enhancement of the defects resulting from inhibiting normal integrin signaling. However, if either of the PAK pathways functions independently of the integrins, a loss of that pathway should enhance the defects caused by inhibiting normal integrin signaling. As reported, we found that tissue-specific expression of the beta tail caused low penetrance defects in gonad morphogenesis (Figure 6F). When these transgenic lines were crossed into the triple *pak-1;pix-1;git-1* mutants or were examined in a *max-2* RNAi background there was no significant enhancement in the defects (Figure 6F). As a control, we also tested whether the beta tail would enhance the defects of a mutant in a pathway that is expected to function independently of integrins. We utilized the UNC-6/UNC-40/UNC-5 pathway which specifically controls the dorsal migrations of the DTCs [12],[13]. As has previously been reported, we found that loss of the *unc-40* gene resulted in defects specifically in the dorso-ventral guidance of the DTCs (Figure 6F). RNAi of *unc-40* in the beta tail transgenic background resulted in additive enhancement of the DTC defects. These results collectively suggest that both the RAC/PAK and the GIT/PIX/PAK pathways function with the integrins to control DTC morphology, migration and guidance.

## Discussion

During gonad morphogenesis, the distal tip cell (DTC) leads the elongating gonad over a long distance to reach its final destination. Several guidance and motility systems are known to facilitate the elongation of the gonad [32]. For example, a protease system that rearranges the ECM allowing motility (GON-1) and guidance (MIG-17) of the DTC are required for proper gonad elongation. Another is the UNC-6/UNC-40/UNC-5 system which specifically directs the dorsal (phase 2) turning of the gonad. Finally there is the integrin system, which controls multiple aspects of gonad elongation by coordinating the interactions between the ECM and the DTC [15],[30],[31]. We have extensively studied the signaling pathways inside the DTC that are regulated by PAKs during gonad morphogenesis, and have identified two distinct PAK signaling pathways that differentially control the morphology, migration and guidance of the DTC. Our analysis also suggests that these PAK pathways are regulated through integrin signaling during gonad elongation.

The two PAK signaling pathways are a classical RAC dependent PAK pathway and a RAC/CDC-42 independent GIT/PIX/PAK pathway. Both pathways function in the guidance of the migrating DTC, but only the latter is required for maintaining the DTC morphology during DTC migrations (Figure S3). What are the roles of PAK-1 and MAX-2 in these two separate pathways? Although our genetic analysis indicates that PAK-1 contributes significantly to the GIT/PIX/PAK signaling pathway, PAK-1 also likely functions in the RAC/CDC-42 dependent pathway. This conclusion comes from the observation that *max-2* single mutants do not yield DTC guidance defects yet double *pak-1;max-2* mutants have profound DTC guidance defects. Therefore a loss of *max-2* is being compensated for by the presence of *pak-1*. However, we also find that double mutants of *max-2;pix-1* or *max-2;git-1* are profoundly defective in guidance even though there is still a functional PAK-1 present. These results suggest that PAK-1 by itself cannot completely compensate for MAX-2 in DTCs. One possible explanation is that *pak-1* is only partially redundant with *max-2*, perhaps due to differential kinase specificity of MAX-2 and PAK-1 while acting as RAC effectors. An alternate interpretation is that the loss of a functional PAK-1/PIX-1/GIT-1 pathway sensitizes the system such that the entire RAC pathway must now remain intact. The latter is supported by our observation that the loss of any component of the PAK/PIX/GIT pathway causes major DTC guidance and migration defects when combined with the loss of any of the *racs* (Figures 1N and 3).

That *git-1*and *pix-1*function together with *pak-1* in a genetic pathway in *C. elegans* strongly supports the notion that these genes have a conserved function across phyla. In addition to our results these proteins have been implicated as working together to regulate cellular processes in diverse model systems. Using genetic analysis in *C. elegans* we demonstrate that this highly conserved GIT/PIX/PAK pathway can function independent of RAC and CDC-42 GTPases. Interestingly, only PAK-1, but not MAX-2, is required, indicating that PAKs are not redundant for this pathway, demonstrating PAK specificity in a RAC/CDC-42 independent pathway. We also attempted to address whether all GTPases are not required in the GIT/PIX/PAK pathway. We generated a mutated PAK-1 that specifically disrupts its P21 binding domain and does not bind to any GTPase, and have found that this mutated PAK-1 can still partially rescue the DTC phenotype in *pak-1* mutants. Our results suggest that perhaps the GIT/PIX/PAK pathway is independent of all GTPases. In addition, our genetic and cell localization studies suggest a model where the GIT/PIX complex is selectively activating PAK-1 through a direct interaction. This conclusion is supported by previous studies in fibroblasts that GIT can activate PAK in the absence of GTPase binding [11]. Furthermore it was recently shown that in T cells a GIT/PIX/PAK pathway functions in parallel to a pathway utilizing VAV (a RAC GEF) along with RAC and PAK [33]. Together, these results suggest that the GTPase-independent GIT/PIX/PAK signaling pathway is a conserved signaling pathway utilized for multiple cellular processes.

In addition to migration defects, the GIT/PIX/PAK pathway mutants exhibit abnormal DTC morphology. Both the migration and morphology phenotypes are consistent with a defect in adhesion to the ECM substrate or the failure to execute coordinated changes in the cytoskeleton. Failure to elongate the proper distance may indicate that the DTCs have difficulty in removing/recycling their contacts with the basal lamina, which could result in the DTCs stalling prior to their targeted final destination. The bloated cell morphology may also result from an adhesion defect. The mutant DTCs may not properly adhere to their substrate and therefore adopt a less organized morphology. Similar DTC phenotypes are also observed in integrin mutants. Regulation of integrin signaling has previously been attributed to the GIT/PIX/PAK pathway in migrating fibroblasts where they are involved dismantling the integrin associated adhesion complexes. Interestingly, orthologs of PAK-1, PIX-1, and GIT-1 are all known to be involved in turnover of focal adhesions [9],[34], and GIT has also been reported to cycle between several different locations including the focal adhesions and cytoplasmic structures [35]. Taken together, it is likely that the GIT/PIX/PAK pathway functions to control either the sorting or the stability of integrin based organization of the cytoskeleton of the migrating DTC.

Our genetic analysis indicates that the two distinct PAK signaling pathways are functioning with the integrins during gonad morphogenesis. First, the overall integrin mutant phenotypes are similar to the combination of mutants from the GIT/PIX/PAK pathway and the RAC/PAK pathway. Second, an interfering construct that is reported to perturb integrin signaling and does cause a gonad phenotype does not significantly enhance the defects of mutants from either of the PAK signaling pathways. Collectively these data support the model that the PAK pathways are all functioning with the integrins. Unfortunately due to the lack of a viable null mutant in any of the integrin subunits our results are less than definitive and there are caveats to our conclusions. First, phenotypic similarity just suggests that they control the same process and does not necessitate that they function together to control that process. Second, the interfering construct causes only weak defects. Because of this we tested whether the construct could enhance an unrelated pathway (UNC-6/UNC-5/UNC-40) and we found that it did enhance this pathway. This clear enhancement of an unrelated pathway strengthens the significance of the non-enhancement with the PAK pathways result and indicates that the interfering construct is likely to disrupt aspects of the integrin signaling pathways that are involved with the PAK signaling pathways. The simplest explanation of our results is therefore that the PAK pathways act with integrin signaling.

It is well known that the RACs are highly redundant for many processes. In *C. elegans* the RACs are only partly redundant. The specific DTC guidance defects in single *rac* mutants (an inappropriate reversal of direction in the final phase of migration) indicate that RAC GTPases are required in a non redundant manner at a specific stage in DTC guidance. It was previously reported that the RACs act with each other to inhibit this extra turn [24]. Such a lack of redundancy in the RAC GTPases may result from RAC specificity at the level of the RACs activator's (the GEFs) or at the level of the RAC effectors. Our results here do not address the redundancy of the RAC GTPases, but they do indicate that any such effector specificity is not occurring through the PAKs (PAK-1 and MAX-2). Instead our results indicate that PAKs are always redundant as RAC GTPase effectors. That is to say either PAK can be activated by any of the RACs. This model predicts that in the case where the RACs are non-redundant either PAK can act with any RAC therefore the PAKs will still be redundant with each other. Similarly if the RACs act together to mediate a pathway the PAKs can both act at either and both steps of the pathway and will still be redundant with each other.

Our conclusion that the two PAKs are completely redundant as RAC effectors comes from multiple lines of evidence. Previously we found that the both PAKs function completely with the RACs to mediate P cell migrations. That is they are completely redundant for this process. However in commissural motor neuron axon guidance *max-2* has a phenotype alone while *pak-1* does not, yet the double is extremely severe (they were partly redundant) [23]. Here we find that the converse relationship is true; *pak-1* has a phenotype alone and the double is very severe. Collectively examining these situations we found that if the *paks* are completely redundant then the individual *pak* mutants do not enhance the individual *rac* mutants. If the *paks* are partly redundant then the PAK with the phenotype would enhance any of the *racs* while the other would not enhance any of them. Our model to account for this describes that the PAKs are redundant as RAC effectors but additional PAK activators exist that do not require RAC GTPases and they activate with specificity towards the PAKs. In P cell migrations there is no such activator, in axon guidance the activator is specific for MAX-2 and during gonad morphology the activator is likely the GIT/PIX complex and it is specific for PAK-1. It is easy to speculate how such a phenomenon could arise evolutionarily. First redundancy at the level of the highly utilized RAC effector pathway would be favorable; after all the RACs themselves are highly redundant and are so in most organisms. This would favor a gene duplication of the PAKs. New roles could then evolve for the PAKs that do not come at a cost of the RAC effector pathway. This would add to the signaling capacity of a cell yet allow it to retain the improved capacity for RAC signaling arising from the gene duplication.

Finally, it is worth noting that the movement of the DTC is distinctly different from the migration of many other migrating cells. Cell migrations are typically characterized by protrusion of filopodia and lamelopodia followed by invasion of the cytosol into these structures, steadily dragging the cell forward. In DTCs we do not observe front protrusion of membranous structures. Instead the migrating DTCs maintain an arrowhead shape during migration (Figure 2), suggesting that they are not moving through a normal fibroblast type mechanism. The DTCs while migrating are also capping a rapidly growing gonad and seem to be pushed from behind by the elongating gonad. Thus the movement of the DTCs is likely to be controlled by the directional secretion of the proteases [17],[21] as well as the regulation of its contacts with the ECM. Our studies indicate that integrin signaling through a novel GIT/ PIX/PAK pathway is important for maintaining the structural integrity and regulating the ECM contacts. Further studies will be necessary to elucidate how these signaling pathways inside the DTC coordinate all these and other factors to properly direct its movement during gonad morphogenesis.

## Materials and Methods

### *C. elegans* and Culture Methods

Worm cultures were maintained with standard methods [36]. All newly characterized mutants were backcrossed at least five times to wild type prior to analysis. Mutant genotypes were confirmed by PCR or direct sequencing of PCR products or by confirmation of a known phenotype. For RNAi experiments, dsRNA was microinjected into the gonad of young adult animals [37]. The following RNAi clones, Ahringer Library Clones [38] unless otherwise specified, were utilized in this study: *max-2* (II 8F19), *pak-1* (C09B8.7 (open biosystems)), *pix-1* (made from the YK clone YK447g6), *ina-1* (III 4N10), *pat-2* (III 4P15), *pat-3* (III 1P02) and *rac-2/3* (IV 7L24).

### The Following Mutant Alleles Were Used in These Studies

LG II: *max-2(cy2)*, *max-2(nv162)*; LG IV: *ced-10(n1993)*, *eri-1(mg366)*; LG V: *rde-1(ne215)*; LG X: *oxIs12[Punc-47::GFP*, *lin-15(+)]*, *pak-1(ok448)*, *pix-1(gk416)*, *git-1(tm1962)*, *mig-2(mu28)*.

### Scoring of DTC Defects and Axon Guidance Defects

To score distal tip cell (DTC) defects, we analyzed young adult hermaphrodites with completely formed vulvas that had yet to pass an oocyte through the spermatheca. For each animal, the anterior and posterior gonads were scored separately. A gonad was deemed to have a DTC defect if the DTC failed to make proper turns (guidance defect), if the DTC failed to reach the vulva (migration defect), or if the DTC had a bloated structure (morphology defect). Specifically, a DTC was deemed to have a guidance defect if it lacked the characteristic U Shape. A DTC was scored as having a migration defect if the DTC was greater than 24 micrometers away from reaching the midline of the vulva. A DTC was deemed to have a morphology defect if the cells diameter (as judged by the diameter of the distal most region of the gonad) was greater than 24 micrometers. The 24 micrometer distance in migration and morphology was chosen as we found that greater than 99% of wild-type animals' DTCs (n = 80) were within this range. For graphical representations these phenotypes were combined and displayed together as the percent of animals with abnormal gonads. The DD and VD commissural motor axon guidance defects were scored as previously described [23].

### Characterization of New Mutant Alleles

The allele *pix-1(gk416)* which was generated by the Vancouver branch of the *C. elegans* Gene Knockout Consortium has a deletion beginning 4 codons after the translational start, which removes the entire SH3 domain and is expected to result in a very early stop codon due to a frame shift. The allele can be followed by the primers 416.f1 gagatacaccccgcaaaaga, 416.f2 gggaaggaacacatgaagga (internal to deletion) and 416.r1 gccgatccacgttgtaaatc. For *git-1* we have utilized the *tm1962* allele generated by Shohei Mitani. *git-1(tm1962)* contains a 484 bp genomic deletion (in frame) resulting in 133 amino acids of the protein being deleted including the second GIT domain. As this domain is required to bind PIX in fibroblasts [9], any functional protein generated in the mutant is expected to be unable to bind PIX-1. The allele can be followed by the primers 1962.f1 ttctccgttgttttcccaag, 1962. f2 gcaccagtatccgaaccacccaa (internal to deletion) and 1962.r1 tagccaatggagatggcatc.

### Tissue-Specific RNAi

For the tissue specific RNAi experiments we expressed an *rde-1* (cDNA) in an *rde-1(ne219)* mutant [26] resulting in a transgenic line (HJ229) that only has functional RNAi where *rde-1* is expressed. To drive the expression of *rde-1* we utilized the *lag-2* promoter (5′ primer ctagacagtcagcggcccataag) up to but not including the start codon and fused this to a *rde-1::unc-54 3′UTR* PCR fragment generated from the pKK1253 plasmid (gift from Hiroshi Qadota).

### Molecular Biology

Cloning of DNA and generation of transgenes were accomplished by standard techniques. In particular we made extensive use of PCR based gene fusion and subsequent cloning of PCR products into TOPO vectors (Invitrogen).

The *Ppak-1::max-2::venus* construct was constructed by fusing the 5′ region of pHJ102 [23] to the 3′ region of the partial cDNA clone *Y38F1A.10::venus* (A gift from Queelim Ch'ng). The resulting construct contained a full length *max-2* cDNA under its own promoter fused to *YFP* (venus). We then fused the *max-2 (cDNA)::venus* region to a *pak-1* promoter [23] to generate *Ppak-1::max-2::YFP*. To generate *Ppak-1::pak-1::mRFP* we generated a *Ppak-1::pak-1(cDNA)* minigene and fused it to the *mRFP::UNC-54 (3′UTR)* from *Punc-25::mRFP* [39] (A gift from Ken-Ichi Ogura). For the PIX-1 translational reporters, we utilized the partial cDNA yk447g6 and fused it to the 5′ *pix-1* genomic region ending at the second exon (5′ primer: gccatggtagtaagagcattccg). This *Ppix-1::pix-1 (cDNA)* minigene was then fused to mRFP or GFP as described in the preceding and following text. To generate *Pgit-1::git-1::GFP* we utilized the yk1688c03 (Yuji Kohara) full length cDNA and fused it to its 5′ genomic region (5′ primer gggtgaacggtcacttgactaga) generating a *Pgit-1::git-1 (cDNA)* minigene. This was then fused to the *GFP::UNC-54 (3′ UTR)* from pPD95.75 (Fire Vector Kit) yielding *Pgit-1::git-1::GFP*. *Lag-2* promoter regions used for DTC specific expression consisted of 2,790 bp of DNA 5′ to the ORF through the start codon (5′ primer acgtcttgtaaccccctcccacc).

### Microscopy

For microscopy animals were mounted on 2% agarose pads with 5 mM sodium azide. Animals were scored by examination with microscopy at 400× on a Zeiss Axioplan II. Confocal images were captured with a Zeiss (Thornwood, NY) LSM 510 META laser-scanning confocal microscope. Images were analyzed using Zeiss META software version 3.2 SPZ.

## Supporting Information

Figure S1 *C. elegans pix-1* and *git-1* are orthologous to fly and human genes and they are expressed in the migrating DTCs. (A–B) A comparison of the percent identity and percent similarity (PI/PS) between conserved domains of *D. melanogaster* (Dm), *H. sapiens* (Hs), and *C. elegans* PIX (A) and GIT (B) orthologs. PIX-1 has significant homology to both mammalian and Drosophila orthologs (A). Humans and mice have two PIX proteins known as α and β. βPIX is highly similar to αPIX particularly in the SH3, RhoGEF and PH domains. The major differences are at its N terminus αPIX contains a calponin domain, while βPIX does not. Neither worm nor fly PIX orthologs contain this calponin motif. For this reason βPIX is used for the comparison. GIT is also highly conserved among worms, flies and humans (B). GIT is characterized by having an Arf GTPase activating (ArfGAP), Ankyrin (ANK) and GIT (also known as Spa2 homology) domains. As with PIX, there are two GIT genes in humans and mice, while a single member is found in flies and worms. The overall domain organizations across these organisms is conserved, however the human GITs each contain three ANK domains while both flies and worms possess two. (C–F) There is significant overlap in the expression of PIX-1 and GIT-1 throughout the development of the animal. Expression from the promoter-GFP constructs starts in early embryogenesis and appears to be present in most cells in the embryo. Expression fads from most cells by late embryogenesis. After hatching the strongest expression is in the pharynx. Expression is also observed in the ventral nerve cord and later in the developing vulva and the DTCs. Fluorescence images from promoter GFP fusions of *Ppix-1::GFP* demonstrate that PIX-1 is expressed in the migrating DTC at early (C) and late larval (D) stages. Fluorescence images from promoter GFP fusions of *Pgit-1::GFP* demonstrate that PIX-1 is expressed in the migrating DTC at early (E) and late (F) stages. The white boxed area in (E) is shown enlarged in the bottom corner. In all figures white arrows point to the vulva and black arrows point to the DTC.
(1.22 MB TIF)

Figure S2 PAK, PIX and GIT tagged proteins are functional and are required in the migrating DTC. Results from transgene rescue assays of the severe gonad morphology double mutant phenotypes. (A) graphical representation of the percent of animals with defects in DTC guidance. *pak-1*, *pix-1* or *git-1* mutants expressing the corresponding rescue transgene under their own upstream promoter sequences were injected with *max-2* dsRNA and both their transgenic and non-transgenic progeny were scored for DTC guidance defects. The n is the number of animals scored. Both the anterior and posterior gonads were scored together for each animal. The GIT-1::GFP and the PAK-1::GFP results are the combination of at least two independently generated transgenic lines, while the PIX-1::GFP results are from a single line. (B) Rescue of mutant enhancement experiments were performed as in (A) except here the described transgenes were under the transcriptional control of the *lag-2* promoter, anterior and posterior gonads were scored individually. To analyze MAX-2::YFP rescue we utilized RNAi with *pak-1* dsRNA. The bars represent the standard error of the mean and asterisks represent significant differences P<0.001. For these experiments all observed lines of the same genotype yielded similar results.
(0.24 MB TIF)

Figure S3 A model for GIT-1/PIX-1/PAK-1and RAC/PAK signaling during DTC migrations. The GIT-1/PIX-1/PAK-1 complex functions in parallel to RAC GTPases and MAX-2 to control distal tip cell late stage migration and distal gonad morphology. In this pathway PAK-1 is activated by the GIT-1/PIX-1 complex independent of GTPases. The GIT-1/PIX-1/PAK-1 complex also contributes to DTC guidance in a manner that is completely redundant with RAC signaling. RAC GTPase signaling through PAKs controls DTC guidance and is partially redundant with the GIT-1/PIX-1/PAK-1 for this process. Both GIT/PIX/PAK and GTPase/PAK pathways function with the integrins to control DTC guidance and gonad morphology. Both MAX-2 and PAK-1 are likely to act redundantly as RAC effectors to regulate DTC guidance.
(0.31 MB TIF)
