# Supplementary figures and images for: A RAC/CDC-42–Independent GIT/PIX/PAK Signaling Pathway Mediates Cell Migration in C. elegans
Source: PLoS Genet. 2008 Nov 21;4(11):e1000269. doi: 10.1371/journal.pgen.1000269 (PMC2581894; doi:10.1371/journal.pgen.1000269)

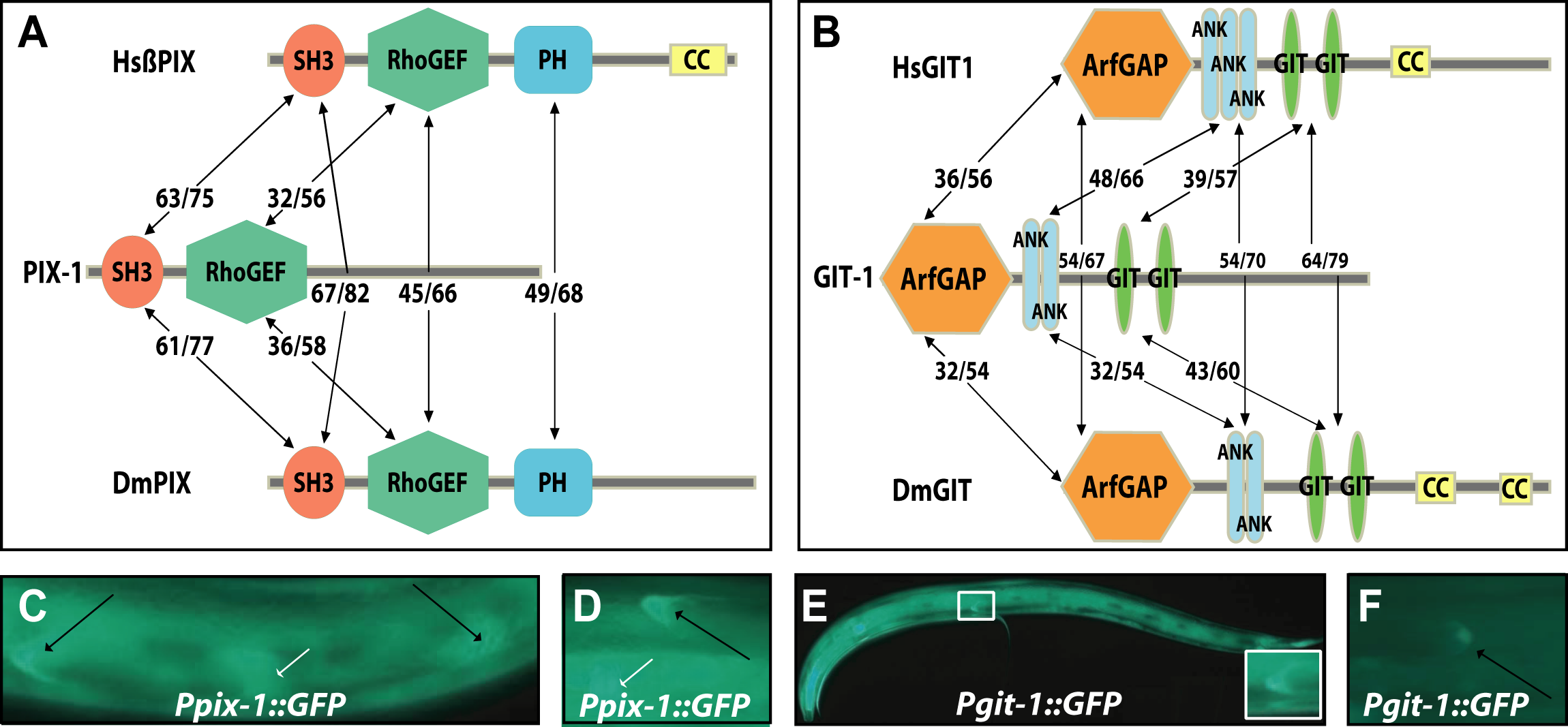

Supplement: Figure S1 — C. elegans pix-1 and git-1 are orthologous to fly and human genes and they are expressed in the migrating DTCs. (A–B) A comparison of the percent identity and percent similarity (PI/PS) between conserved domains of D. melanogaster (Dm), H. sapiens (Hs), and C. elegans PIX (A) and GIT (B) orthologs. PIX-1 has significant homology to both mammalian and Drosophila orthologs (A). Humans and mice have two PIX proteins known as α and β. βPIX is highly similar to αPIX particularly in the SH3, RhoGEF and PH domains. The major differences are at its N terminus αPIX contains a calponin domain, while βPIX does not. Neither worm nor fly PIX orthologs contain this calponin motif. For this reason βPIX is used for the comparison. GIT is also highly conserved among worms, flies and humans (B). GIT is characterized by having an Arf GTPase activating (ArfGAP), Ankyrin (ANK) and GIT (also known as Spa2 homology) domains. As with PIX, there are two GIT genes in humans and mice, while a single member is found in flies and worms. The overall domain organizations across these organisms is conserved, however the human GITs each contain three ANK domains while both flies and worms possess two. (C–F) There is significant overlap in the expression of PIX-1 and GIT-1 throughout the development of the animal. Expression from the promoter-GFP constructs starts in early embryogenesis and appears to be present in most cells in the embryo. Expression fads from most cells by late embryogenesis. After hatching the strongest expression is in the pharynx. Expression is also observed in the ventral nerve cord and later in the developing vulva and the DTCs. Fluorescence images from promoter GFP fusions of Ppix-1::GFP demonstrate that PIX-1 is expressed in the migrating DTC at early (C) and late larval (D) stages. Fluorescence images from promoter GFP fusions of Pgit-1::GFP demonstrate that PIX-1 is expressed in the migrating DTC at early (E) and late (F) stages. The white boxed area in (E) is shown enlar [file pgen.1000269.s001.tif]

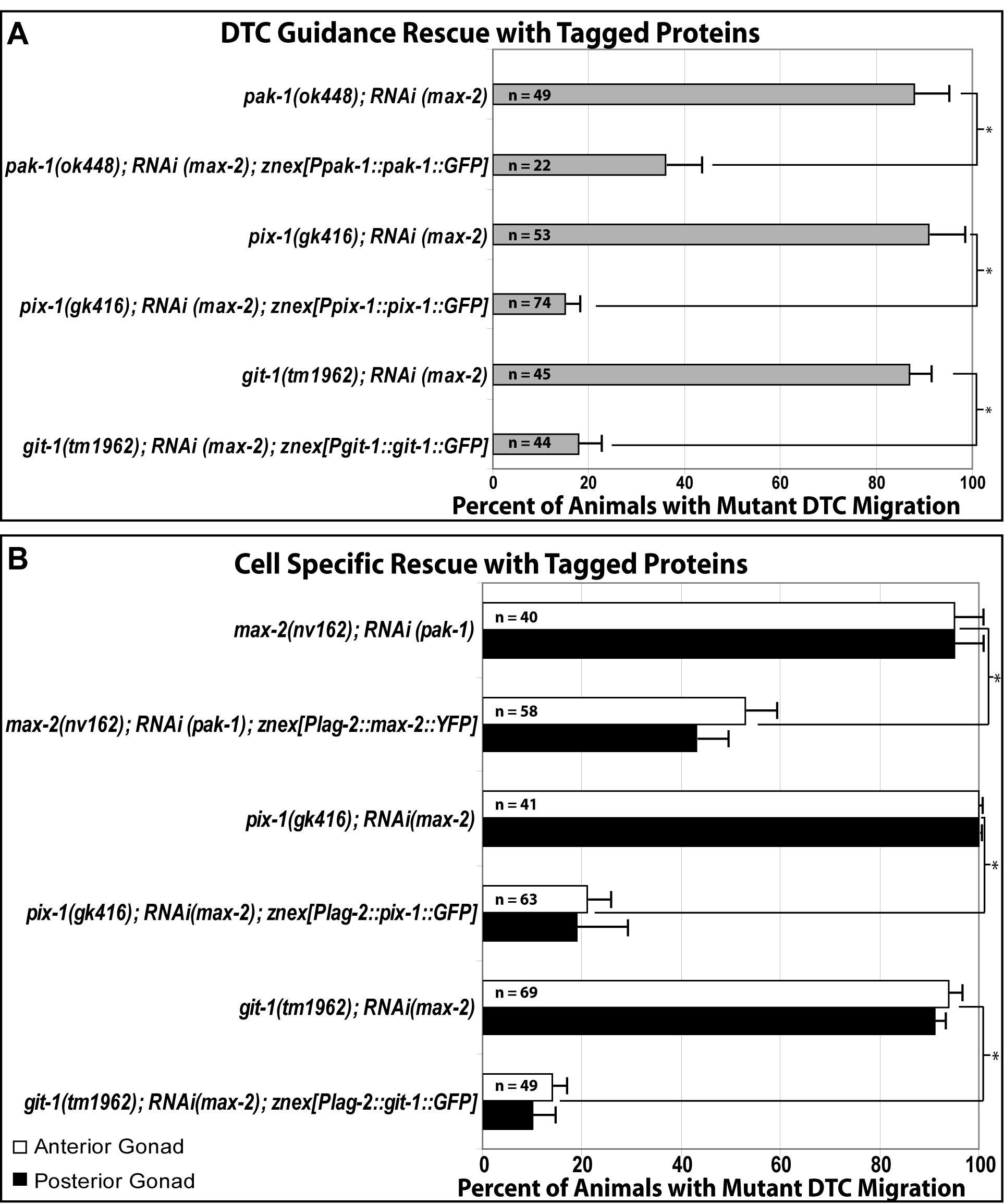

Supplement: Figure S2 — PAK, PIX and GIT tagged proteins are functional and are required in the migrating DTC. Results from transgene rescue assays of the severe gonad morphology double mutant phenotypes. (A) graphical representation of the percent of animals with defects in DTC guidance. pak-1, pix-1 or git-1 mutants expressing the corresponding rescue transgene under their own upstream promoter sequences were injected with max-2 dsRNA and both their transgenic and non-transgenic progeny were scored for DTC guidance defects. The n is the number of animals scored. Both the anterior and posterior gonads were scored together for each animal. The GIT-1::GFP and the PAK-1::GFP results are the combination of at least two independently generated transgenic lines, while the PIX-1::GFP results are from a single line. (B) Rescue of mutant enhancement experiments were performed as in (A) except here the described transgenes were under the transcriptional control of the lag-2 promoter, anterior and posterior gonads were scored individually. To analyze MAX-2::YFP rescue we utilized RNAi with pak-1 dsRNA. The bars represent the standard error of the mean and asterisks represent significant differences P<0.001. For these experiments all observed lines of the same genotype yielded similar results. (0.24 MB TIF) [file pgen.1000269.s002.tif]

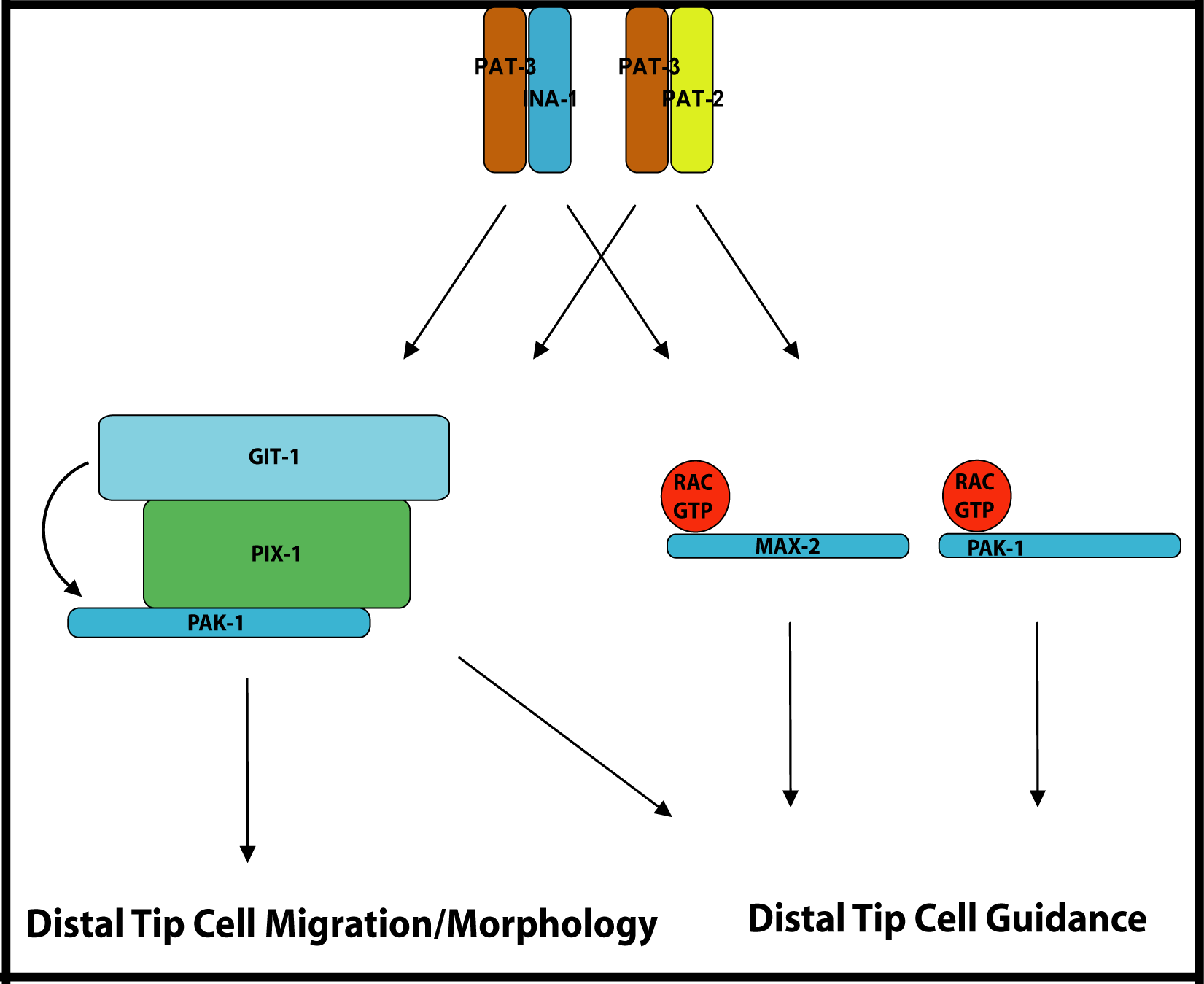

Supplement: Figure S3 — A model for GIT-1/PIX-1/PAK-1and RAC/PAK signaling during DTC migrations. The GIT-1/PIX-1/PAK-1 complex functions in parallel to RAC GTPases and MAX-2 to control distal tip cell late stage migration and distal gonad morphology. In this pathway PAK-1 is activated by the GIT-1/PIX-1 complex independent of GTPases. The GIT-1/PIX-1/PAK-1 complex also contributes to DTC guidance in a manner that is completely redundant with RAC signaling. RAC GTPase signaling through PAKs controls DTC guidance and is partially redundant with the GIT-1/PIX-1/PAK-1 for this process. Both GIT/PIX/PAK and GTPase/PAK pathways function with the integrins to control DTC guidance and gonad morphology. Both MAX-2 and PAK-1 are likely to act redundantly as RAC effectors to regulate DTC guidance. (0.31 MB TIF) [file pgen.1000269.s003.tif]
